# Asphyxia in the Newborn: Evaluating the Accuracy of ICD Coding, Clinical Diagnosis and Reimbursement: Observational Study at a Swiss Tertiary Care Center on Routinely Collected Health Data from 2012-2015

**DOI:** 10.1371/journal.pone.0170691

**Published:** 2017-01-24

**Authors:** Olga Endrich, Carole Rimle, Marcel Zwahlen, Karen Triep, Luigi Raio, Mathias Nelle

**Affiliations:** 1 Medical Directorate, Inselspital, University Hospital of Bern, Bern, Switzerland; 2 Student at the Faculty of Medicine, University of Bern, Bern, Switzerland; 3 Institute of Social and Preventive Medicine, University of Bern, Bern, Switzerland; 4 Department of Obstetrics & Gynecology, University Hospital of Bern, Bern, Switzerland; 5 Neonatology Division, Inselspital, University Hospital of Bern, Bern, Switzerland; Centre Hospitalier Universitaire Vaudois, FRANCE

## Abstract

**Background:**

The ICD-10 categories of the diagnosis “perinatal asphyxia” are defined by clinical signs and a 1-minute Apgar score value. However, the modern conception is more complex and considers metabolic values related to the clinical state. A lack of consistency between the former clinical and the latter encoded diagnosis poses questions over the validity of the data. Our aim was to establish a refined classification which is able to distinctly separate cases according to clinical criteria and financial resource consumption. The hypothesis of the study is that outdated ICD-10 definitions result in differences between the encoded diagnosis asphyxia and the medical diagnosis referring to the clinical context.

**Methods:**

Routinely collected health data (encoding and financial data) of the University Hospital of Bern were used. The study population was chosen by selected ICD codes, the encoded and the clinical diagnosis were analyzed and each case was reevaluated. The new method categorizes the diagnoses of perinatal asphyxia into the following groups: mild, moderate and severe asphyxia, metabolic acidosis and normal clinical findings. The differences of total costs per case were determined by using one-way analysis of variance.

**Results:**

The study population included 622 cases (P20 “intrauterine hypoxia” 399, P21 “birth asphyxia” 233). By applying the new method, the diagnosis asphyxia could be ruled out with a high probability in 47% of cases and the variance of case related costs (one-way ANOVA: F (5, 616) = 55.84, p < 0.001, multiple R-squared = 0.312, p < 0.001) could be best explained. The classification of the severity of asphyxia could clearly be linked to the complexity of cases.

**Conclusion:**

The refined coding method provides clearly defined diagnoses groups and has the strongest effect on the distribution of costs. It improves the diagnosis accuracy of perinatal asphyxia concerning clinical practice, research and reimbursement.

## Introduction

Routinely collected health data (encoded data) are being increasingly used for research purposes. Hospitals in Switzerland are obliged to submit encoded data to the Federal Office of Statistics on an annual basis, enabling publication of epidemiological and economic health care statistics. Both kinds of statistics are influenced by the content and quality of the data. However, encoded data may not be accurate for describing the clinical picture of diseases [1]. This is partly due to the limited updates of the ICD-10 (International Statistical Classification of Diseases and Related Health Problems, Tenth Revision, WHO 1992) resulting in a slow uptake of medical developments over the last 25 years, and the inconsistency between medically determined diagnosis and encoded ICD codes. These inconsistencies affect the accuracy of diagnoses, especially those which refer to clinical signs and symptoms.

To overcome this problem, Switzerland, as well as other countries (e.g. U.S.A., Canada, Australia, France, Germany), elaborated individual coding guidelines, which serve national purposes. The Swiss guidelines are published annually by the Federal Office of Statistics [2]. As ICD codes are being used as selection criteria in epidemiological research, the discrepancy of incidence between different countries might be explained not only by the quality of the health care provider, but also by the national coding guidelines [3]. As a first step, we examined the clinical diagnosis and the ICD code definitions to receive an impression of their disparity.

### ICD-10 definitions

**Fig 1:**
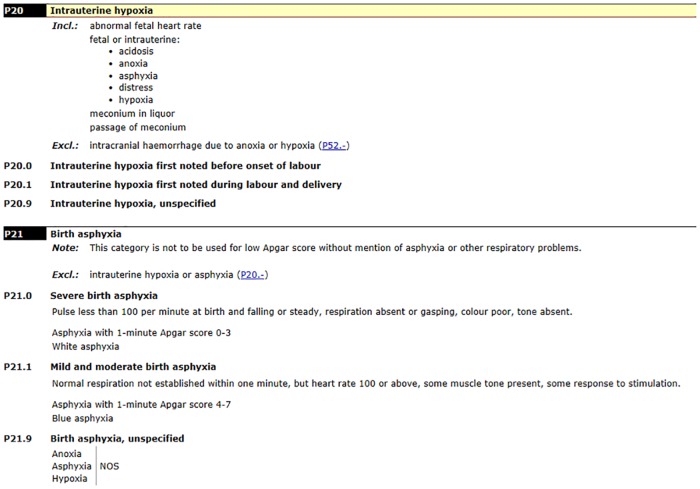
International Statistical Classification of Diseases and Related Health Problems 10th Revision (ICD-10 WHO Version 2016), P20 Intrauterine hypoxia, P21 Birth asphyxia.

The ICD-10 WHO definition of „birth asphyxia”as „failing to initiate and sustain breathing at birth”[4] is specified by the two categories of codes: P20 “intrauterine hypoxia” und P21 “birth asphyxia”, Fig 1. Instead of severity and medical accuracy, the categories are classified by “onset characteristics” (intrauterine versus birth asphyxia). The code P20 “intrauterine hypoxia” has broad inclusion terms and manifestation properties (symptoms) but lacks clear definition and criteria (e.g. „abnormal fetal heart rate“, „distress”), diagnostic criteria and threshold values (e.g. „acidosis“, „anoxia“, „asphyxia“, „hypoxia“) as well as a correlation with the clinical state (e.g. „meconium in liquor“, „passage of meconium“).

The codes of category P21 “asphyxia” are defined in the ICD-10 by the 1-minute Apgar score and additionally by some of the individual elements of the 1-minute Apgar score, which are meant to reflect the severity of mild, moderate and severe asphyxia (heart rate less than 100 or above, impairment of respiration, colour, tone).

Additionally, the exclusion term “Excl.: intrauterine hypoxia or asphyxia (P20.-)” affects the subcategory of the P21 codes, providing rules for the interrelation of codes [5].

### Definition of clinical term

The clinical definition of the term “birth asphyxia” on the other hand has changed over the past 20 years from „failing to initiate and sustain breathing at birth”to „intrapartum-related hypoxia“. Both definitions are complex and open to interpretation [6]. Anne CC Lee [7] classifies measures of intra-partum-related hypoxia into three clusters of terms: 1) process-based indicators (i.e., measures of abnormal obstetric processes), 2) clinical sign-based indicators (i.e., low Apgar scores, fetal acidosis), and 3) outcome-based indicators (i.e. fetal-neonatal mortality or morbidity). Previously, symptom-based indicators, such as the Apgar score, were commonly used to define “birth asphyxia”. Here we note an obvious inconsistency between diagnosis criteria concerning ICD-10 code and medical diagnosis. It is probable that signs such as „abnormal fetal heart rate”or „meconium in liquor”would be encoded as P20.1 “intrauterine hypoxia”, without the clinical diagnosis “hypoxia” being present itself.

Severe asphyxia is associated with multiple organ failure including hypoxic encephalopathy [8]. The American Academy of Pediatrics (AAP) and the American College of Obstetrics and Gynecology (ACOG) encourages the term „neonatal encephalopathia”(NE) instead of „hypoxic-ischemic or post-asphyxial encephalopathia“, except where injury by intrauterine hypoxia is highly probable [9]. There has been intensive research between 1980–2000 worldwide, such as clinical trials in systemic hypothermia, attempting to define the term „hypoxic-ischemic encephalopathia (HIE)”which is still in use in Switzerland [10–21]. For the past 15–20 years systemic hypothermia has been established as the standard treatment regimen in HIE [10, 11], the degree of severity being determined according to Sarnat & Sarnat [22]. The criteria of indication for therapy meet those of severe asphyxia, as described by Jacobs SE, „Cooling for newborn, Cochrane-Review 2013”[11]–and can be summarized as follows: evidence of peripartum asphyxia within 60 minutes of birth as determined by the Apgar score, mechanical ventilation or resuscitation, cord or arterial pH, base deficit; evidence of encephalopathy according to Sarnat staging. From 2010 onwards, it has been possible to encode the diagnosis HIE in Switzerland by ICD-10 code P91.6 “hypoxic-ischemic encephalopathia“. Further diagnoses P91.3 „neonatal cerebral irritability“, P91.4 „neonatal cerebral depression”and P91.5 „neonatal coma”were assigned to neurological signs and symptoms according to Sarnat. From then on, systemic hypothermia has also been encoded specifically by code 99.81.20 „systemische Hypothermie”(i.e. systemic hypothermia; Swiss classification of procedures CHOP (Schweizerische Operationsklassifikation) 2011 [23].

### Reimbursement regulations

The different definitions (classificatory and clinical) not only influence the quality and comparability of statistical reports but also have a high impact on reimbursement of hospital services in inpatient care.

Since 2012, the federal law of reimbursement for acute inpatient care SwissDRG (Swiss Diagnosis Related Groups) has been based on payment rate-setting mechanisms using standardized cost data and classification into diagnosis-related groups [24].

Mandated by federal law the CMO (Case Mix Office) SwissDRG calculates annual “case-based” or “case-mix-based”rates of the DRG price and reimbursement using both encoded data (ICD, CHOP) and patient-level costs through regression models. This allows classification of patients into clinically meaningful groups which consume similar health-care resources. Different diagnoses are likely to result in significantly different resource consumption (complication and co-morbidity level CCL; 0–4) during an inpatient episode. DRGs have differing levels of resource consumption and are split on the basis of CCL or on the basis of certain functions (e.g. “schweres Problem”, engl. “severe problem”), thus, presenting a certain relative weight (= severity weight).

The inconsistencies between the definitions of the clinical diagnosis and ICD code cause an inadequate assignment of costs and resources, especially with regard to the severity of disease.

### Designing the “Model Matrix” method

In acknowledgment of inconsistencies in the correct allocation of resources corresponding to the severity of the diagnosis, the Federal Office of Statistics assimilated the coding guidelines upon the request of the authors/University Hospital of Bern. The application was put forward in 2014, revised by advisory boards and implemented in 2016 (Coding Guidelines 2016; model „KHB.2016“), [25]. S1 Fig. Subsequently a refined coding guideline was elaborated in 2015 to separate all characteristics of peripartum hypoxia by neurological signs /symptoms and biochemical values, which allowed to classify the cases distinctly by clinical state and metabolism (“Model Matrix”).

The refined guidelines were based on standard values from literature, e.g. a low 5-minute Apgar score as a high-risk marker in association with severe fetal acidemia or intubation within the first hour of life as highest risk for developing seizures secondary to perinatal asphyxia [26, 27]. By applying the model “Model Matrix” this method allowed the authors to outline the severity of perinatal neurological and metabolic impairment since etiology and time of occurrence (intrauterine long-term, intrauterine peripartum) are not bound to hard criteria by hypothesis. The „Model Matrix”was designed referring to the most important RCTs (randomized controlled trial) concerning treatment by hypothermia. In order to enable the encoding of a metabolic value (acidosis) lacking neurological complications, definitions were attributed to code P20. We analyzed and validated encoded data and case related costs.

Our aim was to specify the ICD definition of perinatal asphyxia and to develop a refined method of an accurate encoding of the diagnosis perinatal asphyxia Thus the refined coding method improved the accuracy of diagnosis of perinatal asphyxia concerning clinical practice, research and reimbursement.

## Materials and Methods

### Data

The University Hospital of Bern-Inselspital is a tertiary care center, specializing in gynecology and obstetrics, pediatrics incl. PICU (pediatric intensive care unit), neonatology incl. NICU (neonatal intensive care unit) and NIMC (neonatal intermediate care unit) and pediatric surgery providing both in- and outpatient care.

Shortly after discharge from hospital the inpatient case is encoded by medical coding specialists based on the information received from the electronic medical record. The data (routine data / health administration data) has to be submitted for the reimbursement process (SwissDRG) and in-house and national statistics (Medizinische Statistik der Krankenhäuser) whereby it passes several quality checks. Case related costs are recorded according to REKOLE^®^ [28], the standard costs accounting system available in the hospital’s business data warehouse.

**Fig 2:**
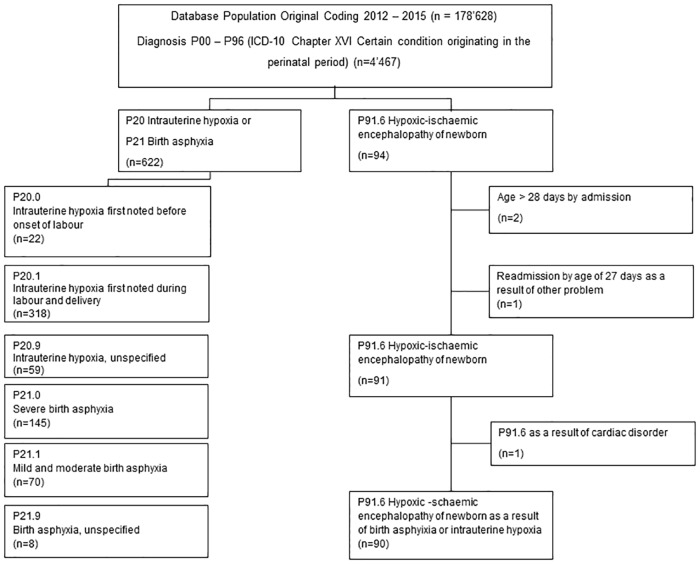
Flow chart of the selection process.

The International Statistical Classification of Diseases and Related Health Problems ICD-10 German Modification (GM) [29] codes were used to encode main and secondary diagnoses in the medical statistic (MS) data set. In regard to the codes of diagnoses asphyxia and HIE the catalogues ICD-10 WHO and ICD-10 GM used for coding in Switzerland are identical. The ICD-10 codes P20 and P21 were chosen to select the patients with birth asphyxia treated from 2012–2015 at the University Hospital of Bern. In order to exclusively analyze newborn cases, patients older than 28 days upon admission were excluded from the datasets. Eleven patients had both P20 and P21 codes in one record, hence only one diagnosis was chosen according to the Swiss Coding Guidelines of the year of admission. After applying these restrictions, a total of 622 neonatal inpatients were identified, 452 of them inborn, and 170 outborn. The group of newborns with HIE due to perinatal asphyxia were selected by using the code P91.6, and consisted of 90 cases, Fig 2.

The encoded medical data and the data of case related costs are linked in a QlikView database, and clinical data (excel database) are manually linked through case identification number. In order to assess incidence, this data is compared to the national statistics provided by the Federal Office of Statistics.

To derive information on the course of treatment administered per patient, all medical records were analyzed (first and second author). The information on treatment procedures was encoded according to the CHOP (28). We classified treatment into three groups based on available information: 1) CHOP codes which indicated mechanical ventilation; 2) CHOP codes which indicated a systemic hypothermia; and 3) CHOP codes which indicated significant OR (operation room) procedures (S1 File).

The quality of encoded data was validated by evaluating the accuracy of the determined codes with the coding guidelines of admission year (first author). The values of biochemical analyses, neurological scores and clinical diagnosis were extracted (second author) using case ID (case identification number) as a unique identifier. The datasets were anonymized.

The medical statistic dataset included information on encoded diagnosis, procedures, gestational age, birth weight, age at admission (outborn), weight at admission (outborn), length of stay and DRG.

The clinical information system included variables such as medical diagnosis, Apgar score at 1, 5 and 10 minutes of age, Sarnat stage, HIE therapy, biochemical values in the first hour postpartum, lowest values of pH (umbilical artery, umbilical vein, blood), BE (umbilical artery, umbilical vein, umbilical artery standardized, umbilical vein standardized, capillary standardized), lactate (umbilical artery, umbilical vein, capillary). The values of inborn newborns were extracted from electronic laboratory records, the values of outborn patients from admission letters. Unavailable data were labeled as ‘missing value’.

### Recoding

Automated coding was created through excel macro demonstrating cut- off values of Apgar score, pH, BE, lactate und Sarnat stage per model with regard to the previously defined criteria per case. The corresponding diagnosis was encoded once a minimum of criteria was fulfilled. The relevant variables and values were documented. The excel macro was manually checked at random sampling, comparing the results to those of the automated excel macro, being identical in 100% of the cases. The cases with HIE and hypothermia served as control group.

### Model criteria

The following models were both defined based on previously existing coding guidelines (effective from 2012–2016): „Original Coding”= originally encoded by the catalogues being used at that time and coding guidelines (ICD-10, Swiss Coding Guidelines 2012–2015), and „KHB.2016”= encoded by catalogues and Swiss Coding Guideline (KHB) used in 2016.

**Fig 3:**
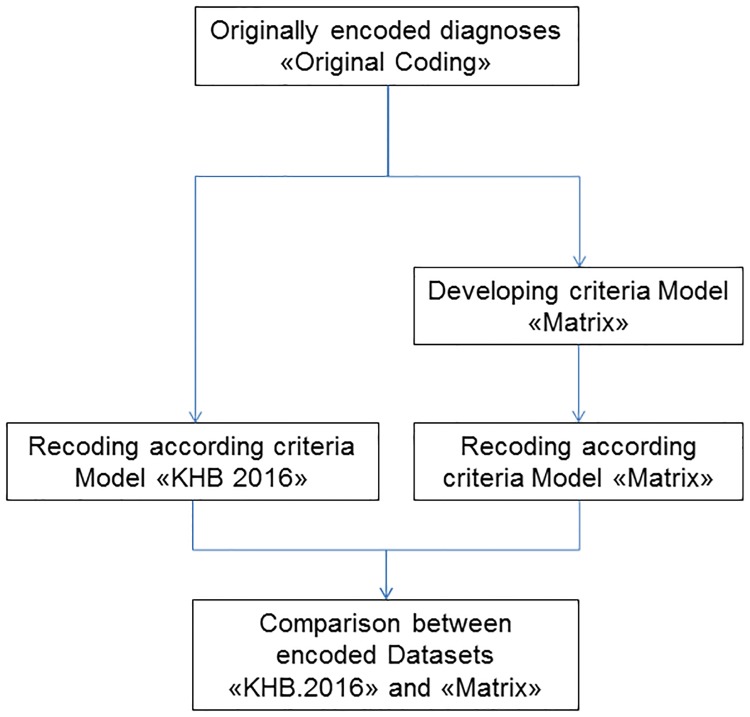
Development process of the model “Model Matrix”.

Criteria of model “KHB.2016” were verified by identifying both advantages and disadvantages with respect to a consistent classification of all cases. By encoding according to the results of this analysis, the criteria of the newly defined model (“Model Matrix”) were adapted, Fig 3.

The most important RCTs concerning hypothermia treatment and national and international criteria of indication were reviewed and taken into consideration when defining „Model Matrix“. Table 1 shows the criteria of indication for hypothermia treatment of the relevant RCTs, including mean values, SD values in Control and Trial Groups.

**Table 1:** Including criteria Hypothermia, incl. Mean, SD of reported values, Review.

Study / Guideline	Apgar 1 min	Apgar 5 min	Apgar 10 min	pH	BE	lactate	Mech. Vent (min)	Resusci-tation
Jacobs 2003			< = 5	< 7.1	<-12			
Akisu 2003		< 6 (mean, SD: TG1^a^ 4.1, 1; TG2 4.3, 1)		< 7.1 (mean, SD: TG1 7.03, 0.1; TG2 7.02, 0.1)	<-10 (mean, SD: TG1–15.3, 8; TG2 14.2, 10.2)			
Cool Cap Study 2005			≤5		<-16			> 10 min
Eicher 2005		(Mean, SD: 5, 2)	≤5	< 7 (mean, SD: 6.95, 0.19; 6.96, 0.23)	<-16 (mean, SD: -18, 8.3; -16, 7.5)			>5 min
Gunn 1998		≤6 (mean, SD: CG^b^ 4.5, 2; TG 4.7, 2; TG 6.0, 1)		≤7.09 (mean, SD: CG^a^ 6.79, 0.25; TG^b^ 6.98, 0.21; 6.93, 0.11)				
ICE Study			≤5	≤7 (mean, SD: 6.9, 0.2)	≤-12		>10	
Lin 2006			<6	<7.1		>15		
Neo.nEURO Study 2010		(mean, SD: CG 3.4, 2.4; TG 3.2, 2.4)	<5	<7 (mean, SD: CG 6.9, 0.2; TG 6.9, 0.2)	≤-16 (mean, SD: CG 19.5, 6.8; TG 19.4, 6.2)		>10	
NICHD Study 2005				≤7, if no blood gas or pH 7.01 to 7.15 or BE 10 to 15.9 additional criteria required; (mean, SD: TGa 6.8, 0.2; TGb 6.9, 0.2)	≤-16 (mean, SD: 20.6, 7.5; 17.5, 7.7)			
Shankaran 2002				≤7, pH 7.01 to 7.15 or BE 10 to 15.9 additional criteria required; (mean, SD: TG1 6.94, 02; TG2 6.95, 0.2)	≤-16 (mean, SD: TG1 16.2, 7.8; TG2 15.9, 6.7; TG3 16.1, 7.5; TG4 16.0, 7.0)			
TOBY Study 2009			<5	<7 (mean, SD: 6.9, 0.2)	<-16		>10	
Zhou 2010	≤3	<5		<7	≤-16		yes	>5 min
Guidelines Swiss Society of Neonatology				<7 and Sarnat II-III	<-16			
Guidelines Bern University Hospital Pediatric ICU		<6	<6	<7 and Sarnat II-III	<(12)16		>(5)10 min	

^a^TG: Trial Group.

^b^CG: Control Group

By refining the Coding Guidelines of 2016, the group classifications of mild, moderate and severe asphyxia diagnosis in P21, as well as the identification of further clinical states to be included in the P20 encoding were made possible. Certain definitions were assigned to code P20 in order to include the encoding of metabolic values (acidosis) lacking neurological complications. The newly classified diagnosis groups were analyzed (mean value, SD, ranges) in respect to each model, corresponding to an overview of criteria for hypothermia.

Certain criteria of model “KHB.2016”, such as „necessity of intensive care treatment in neonatal intensive care unit”or „ventilation”which did not adequately correspond to a diagnosis, but rather reflected an issue of infrastructure or a behavioral intention, were not considered for the revision.

As there is no clearly established distinction between mild and moderate asphyxia, the low cut-off value was based on a review of patient records and audit case study and a mean ±SD pH of all newborn between pH 7.15 and 7.20 was calculated defining the low cut-off value at pH of 7.15 [15, 19].

As a result of the analysis the „Model Matrix”was developed.

### Criteria according to „Model Matrix“

Diagnoses of category P21.- birth asphyxia can be encoded when fulfilling the following criteria (even, if the term „asphyxia”is not mentioned explicitly):

#### P21.0 severe birth asphyxia

At least 3 of the criteria mentioned below must be fulfilled:

- 5-minute Apgar score ≤ 5
- severe acidosis during first hour of life: pH ≤ 7.00 (UV, UA, capillary or arterial blood sample)
- basedeficit ≤ -16 mmol/L in UV or UA or during first hour of life
- moderate to severe encephalopathy (Sarnat stage II—III)
- lactate ≥12 mmol/L in UV or UA or during first hour of life

#### P21.1 moderate birth asphyxia

At least 2 of the criteria mentioned below must be fulfilled:

- 5- minute Apgar score ≤ 7
- moderate acidosis during first hour of life: pH < 7.15 (UV, UA, capillary or arterial blood sample)
- mild to moderate encephalopathy (Sarnat stage I—II)

#### P21.9 mild asphyxia without metabolic acidosis

Both of the two criteria mentioned below must be fulfilled:

- 5- minute Apgar score ≤ 7 and
- lowest value 1 hour of life pH ≥ 7.15 (UV, UA, capillary or arterial blood sample)

#### P20.1 metabolic acidosis without neurological impairment

Metabolic acidosis without clinical impairment (i.e. asphyxia)

- 5- minute Apgar score > 7
- moderate acidosis during first hour of life: pH < 7.15 (UV, UA, capillary or arterial blood sample)

#### Norm

- 5- minute Apgar score > 7
- Lowest value 1 hour of life pH ≥ 7.15 (UV, UA, capillary or arterial blood sample)

The originally encoded diagnoses were adjusted manually in the dataset of medical statistic MS [2] according to the recoding by excel macro. Three MS datasets were created: „Original Coding“, „KHB.2016“, „Model Matrix“.

### Statistical analysis

The datasets were regrouped using batch grouping [30], revenues were simulated according to the version of SwissDRG and the results of the three models were compared. The data (total costs, earning AP DRG, SwissDRG) were tested for normality and equal distribution graphically and were assessed for skewness and kurtosis using Shapiro-Wilk test. Between-group comparisons („Original Coding“, „KHB.2016“, „Model Matrix“) were performed with one-way analysis of variance (ANOVA), means and standard deviations (SDs) were calculated for continuous variables (total costs (real number, log10), earning (real number, log10)) with Levene’s test for homogeneity. P < 0.001 being considered as statistically significant. Descriptive statistic and graphic were used to test for differences in patients with clinical findings. The Revenue (“Income SwissDRG”, SwissDRG Version), the outliers (“high deficit”, “high profit” cases) and high deficit per diagnosis group per model were calculated. All statistical analyses were performed using R software.

### Software

Medical Coding Software SAP IS-H Klinischer Arbeitsplatz, Medical Coding Tool ID Diacos, Clinical Data Phoenix CGM, Business Data WareHouse SAP BW, Microsoft Excel 2010, R (www.r-project.org, packages ggplot2, car, pastecs, Rcmdr), Notepad++, v. 6.7.5, free software.

### Ethics

The Ethics Committee of the Canton Bern approved our study (KEK-Nr. Req-2016-00025). Informed consent was not necessary, as the analyses were done with routine clinical and financial data from our hospital for quality assurance purposes.

## Results

### Incidence

The number of diagnoses P20, P21 and P91 in Switzerland as well as the number of births from 2004–2014 were derived from the national statistic (Medizinische Statistik der Krankenhäuser) and incidence was calculated (S1 and S2 Tables).

The resulting incidence for diagnoses P20.-, P21.- and P91.6 is shown in Table 2.

**Table 2:** Incidence of P20* and/or P21* Intrauterine hypoxia and/or birth asphyxia, P91.6 Hypoxic-ischemic encephalopathy of newborn per 1000 live births, Medical Statistics, Swiss Federal Statistical Office.

Incidence per 1000 live births	Year										
	2004	2005	2006	2007	2008	2009	2010	2011	2012	2013	2014
Live Births, MS^a^	71430	72193	72946	73989	76212	77690	80508	80646	82607	83098	85234
P20* and/or P21* Intrauterine hypoxia and/or birth asphyxia	56.12	80.17	77.93	80.31	76.51	71.51	58.43	59.53	56.39	48.91	44.24
**P21.0 Severe birth asphyxia**	4.7	6.43	6.37	7.51	8.69	8.42	7.95	8.43	8.28	7.05	5.63
**P91.6 Hypoxic-ischemic encephalopathy in the newborn**						1.004	0.732	1.078	1.15	1.01	1.197
(P20*, P21* or P91*) and procedure 99.81.20^b^								0.632	0.423	0.613	0.798

^a^The number of live births according MS (Medical Statistics of the Hospitals): included only the births in hospital-setting

^b^The (P20*, P21* or P91*) and procedure 99.81.20 as equivalent of “Hypothermia by intrauterine hypoxia, birth asphyxia or disturbance of cerebral status of newborn”. P20*, P21* or P91* only as a main diagnosis by newborns, age by admission < 6 days.

### Patient characteristics

Of the 622 cases 452 were inborn, and 170 were outborn. The mean length of stay was 15.31 days (min 1, max 249), mean gestational age 37 5/7 weeks (min 24 3/7, max 43 0/7), mean birth weight 2870 g (min 535 g, max 4990 g). HIE was diagnosed in 98 newborns, Sarnat Stage documented as I, II, III in 31, 44, 23 newborns respectively. 63 newborns were treated with hypothermia, of which 55 were outborn (10.1% of all newborn, 32% of outborn). 227 patients received mechanical ventilation or CPAP (continuous positive airway pressure), 25 patients underwent significant OR procedures.

Clinical and biochemical findings such as Apgar score are shown in Table 3. The number of missing pH values add up to only 5 cases, because either the umbilical venous (UV), arterial (UA), capillary or venous blood pH is measured, the missing BE values add up to 100 cases (UV, UA, capillary or venous blood) and the missing lactate values (UV, UA, capillary or venous blood) to 316 cases, S1 Data

**Table 3:** Clinical values within 60 min after birth (2012–2015, n = 622).

Clinical values	Min	1Q	Median	Mean	3Q	Max	MissingValue
**Apgar at 1 min of age**	0	2	5	4.95	8	10	5
**Apgar at 5 min of age**	0	5	7	6.767	9	10	5
**Apgar at 10 min of age**	0	7	9	7.857	9	10	5
**UA pH**	6.5	7.08	7.179	7.162	7.280	7.440	161
**cap ven pH**	6.52	6.96	7.08	7.084	7.207	7.470	406
**UA BE**	-29.0	-10.0	-6.0	-6.358	-2.0	4.0	274
**UV BE**	-17.0	-7.0	-4.0	-4.746	-1.0	3.5	313
**UA BE standard**	-28.0	-7.0	-3.2	-4.134	0.0	4.5	321
**UV BE standard**	-25.0	-6.9	-4.0	-4.352	-1.0	4.2	311
**BE cap standard**	-29.0	-13.4	-8.35	-9.280	-3.525	2.3	476
**BE ven standard**	-27.4	-12.5	-7.8	-8.6	-4.8	1.8	489
**Lactate cap**	0.9	3.45	6.0	7.889	11.7	24.0	467
**Lactate ven**	1.4	4.6	7.2	8.296	11.7	25.0	465

UA, umbilical artery; UV, umbilical vein; cap, capillary; ven, venous; BE, base excess; standard, test standardized

### Economic data

The case related total costs were calculated by the national cost accounting method REKOLE^®^ [28]. Income SwissDRG was calculated based on the effective case weight classified by SwissDRG catalogue version 1.0 (2012), 2.0 (2013), 3.0 (2014), 4.0 (2015) according to year of discharge and multiplied by the corresponding base rate (11’425 CHF in 2012, 11’200 CHF in 2013, 11’000 CHF in 2014, 11’000 CHF in 2015). The additional payments (“Zusatzentgelt”) were determined according to the SwissDRG version [24], the profit being the difference between Income SwissDRG (revenues incl. additional payments) and total costs (Table 4).

**Table 4:** Total costs and Income SwissDRG in 2012–2015, (n = 622).

	Study Cases, (n = 622)	Mean	Min	Max	SD
**Total Costs REKOLE**^**®**^^a^, **CHF**^b^	20‘612‘100.9	33‘138.42	1‘031.92	699‘689.95	61‘447.18
**Income SwissDRG (incl. additional payments**^c^), **CHF**	19‘780‘759 (56‘161)	32‘138.95	1‘268.18	661‘907.4	58‘966.83
**Profit SwissDRG, CHF**	-831‘341.9				

^a^ REKOLE^®^, standard method for costs accounting in Swiss Hospitals.

^b^ CHF, swiss franc, currency rate at 01.01.2016: 1 CHF = 0.999 USD.

^c^ additional payments, “Zusatzentgelte”.

### Recoding

**Fig 4:**
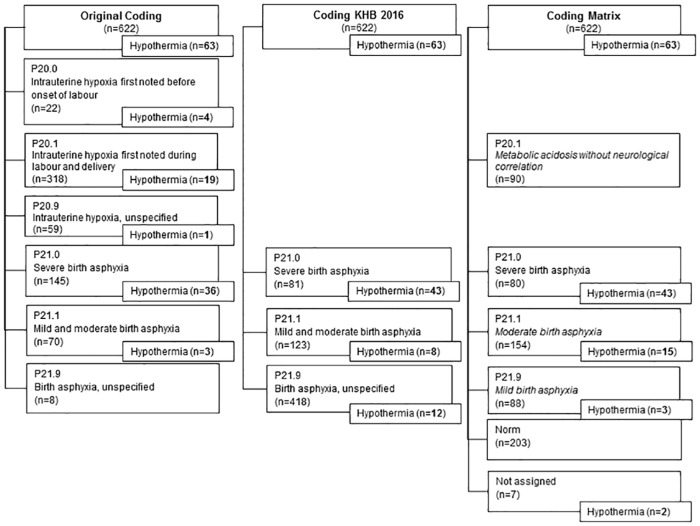
Distribution of cases after recoding.

The classification of the cases into distinct DRGs changed by revision of the encoded diagnoses, see flow chart Fig 4 for distribution of cases.

#### Recoding original coding according to criteria “KHB.2016”

Table 5 shows the results of mapping the cases from group „Original Coding”to „KHB.2016“. Of the 22 cases originally encoded with diagnosis P20.0 only 6 (27%) fulfilled the criteria of severe asphyxia according to “KHB.2016”. 246 cases originally encoded with code P20.1 (77%) were changed to P21.9. Of originally encoded severe asphyxia (P21.0) only 41 cases (28%) remained in the same group. Overall 67% (418 cases) of all cases with the original diagnosis of P20 und P21 had to be reallocated into the group P21.9 „birth asphyxia unspecified“.

**Table 5:** Recoding Original Coding according criteria KHB.2016 (n = 622).

	P20.0.orig (n = 22)	P20.1.orig (n = 318)	P20.9.orig (n = 59)	P21.0.orig (n = 145)	P21.1.orig (n = 70)	P21.9.orig (n = 8)
P21.0.KHB.2016 (n = 81)	6	25	3	41	3	3
P21.1.KHB.2016 (n = 123)	5	47	22	38	9	2
P21.9.KHB.2016 (n = 418)	11	246	34	66	58	3

#### Recoding original coding according to criteria “Model Matrix”

The originally encoded diagnosis P20.0 showed a heterogeneous distribution to all groups from severe asphyxia to normal clinical finding (Table 6). Most of the cases of P20.1 had to be mapped to „Norm”(142 cases, 45%) or to P20.1 “metabolic acidosis without neurological impairment”(64 cases, 20%), in total 65% (206 cases) of originally encoded diagnosis P20.1. 41 cases (30%) of severe asphyxia (P21.0) remained in the same category.

**Table 6:** Recoding Original Coding according criteria Matrix (n = 622).

	P20.0.orig (n = 22)	P20.1.orig (n = 318)	P20.9.orig (n = 59)	P21.0.orig (n = 145)	P21.1.orig (n = 70)	P21.9.orig (n = 8)
P20.1.matrix (n = 90)	0	64	13	4	9	0
P21.0.matrix (n = 80)	6	25	3	41	3	2
P21.1.matrix (n = 154)	7	57	23	55	9	3
P21.9.matrix (n = 88)	1	27	4	33	21	2
Norm.matrix (n = 203)	8	142	15	9	28	1
Not.assigned.matrix (n = 7)	0	3	1	3	0	0

### Comparing encoding by “KHB.2016” to “Model Matrix”

**Fig 5:**
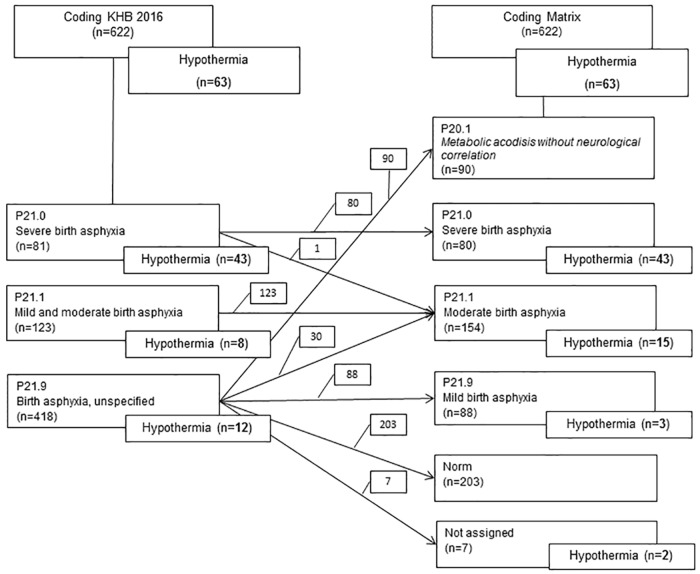
Distribution of cases comparing “KHB.2016” to “Matrix”.

Fig 5 presents the adjustment of cases by encoding according to „Model Matrix”instead of „KHB.2016“.

#### P21.0 severe asphyxia (KHB.2016, Matrix)

The diagnosis P21.0 „severe asphyxia”encoded according to “KHB.2016” and “Model Matrix” showed the same results. The cases were identical, except for one case (KHB.2016 n = 81, Model Matrix n = 80) which was allocated in group P21.1 “Model Matrix” because of the 10 min Apgar score (criterion in Model “KHB.2016” but not in “Model Matrix”; Apgar score 7/7/2). 43 of patients in group P21.0 „KHB.2016”and „Model Matrix”received systemic hypothermia treatment (68% of total SH).

#### P21.1 mild and moderate asphyxia (KHB.2016), moderate asphyxia (Matrix)

30 cases of group P21.9 encoded according to „KHB.2016”could be mapped to group P21.1 “Model Matrix” (including 7 with treatment of hypothermia).

The reason for this relevant reallocation is the threshold value of the Apgar score and consideration of the Sarnat stage. 10 cases showing an Apgar score < 4, but pH < 7.15 were allocated in group P21.9 “KHB.2016”, as the Apgar score was “too low”. Another 11 cases with Apgar score > 3, UA pH < 7.15 and Sarnat stage I-II were mapped to P21.1 “Model Matrix”. The crucial factor was the significance of the Sarnat stage, which has no criterion in model „KHB.2016”regarding diagnosis P21.1, resulting in an allocation of cases with moderate asphyxia into group P21.9.

By refining the model „KHB.2016”more specific diagnoses were applied to 30 cases of group P21.9 „birth asphyxia unspecified”(7 of them received treatment of systemic hypothermia), i.e. P21.1 „moderate asphyxia”according to model „Model Matrix“. By setting a low cut-off value for the Apgar score minimum of 4 for the diagnosis P21.1 according to “KHB.2016”, patients with low values (e.g. Apgar 0 and pH 7.15) were excluded and classified into the diagnosis group P21.9.

#### P21.9 asphyxia unspecified (KHB.2016), mild asphyxia (Matrix)

Group P21.9 shows only 88 cases when encoded according to „Model Matrix”(418 “KHB.2016”), 54% of them preterm (48/88), which corresponds to 26% of all preterm patients at a GA < 37 WGA (48/183 total). 34% of cases P21.9 „Model Matrix”were born at a low birth weight < 2000 g (30/88), of these 10 < 1000 g (31% of all cases < 1000 g).

#### Not assigned (Matrix)

This group consists of 7 high complexity cases, 3 of them unattended home births / non-clinical setting with no biochemical values available 1h pp, 2 of these cases were preterm births born at a low birth weight of 1815g and 700g and 1 case was a term newborn; 3 cases were transferred from other hospitals (suspected asphyxia, no biochemical values available 1h pp); 1 patient with suspected infection and transfer for surgical delivery (emergency caesarean section, born at a low birth weight of 850g, no blood sample 1h, resuscitation); 3 of 7 cases were born at term, 2 of them received hypothermia treatment, 4 did not as they were preterm.

#### Normal clinical finding (Matrix)

Most cases (45% of „normal clinical finding“) were originally encoded in group P21.1 (142 of 318). 28 cases (40% of P21.1 originally encoded) were allocated to “normal clinical finding” (1- minute Apgar score 4–7), resulting in 14% of group “normal clinical finding”.

### Biochemical values

Analysis of the Apgar, pH, BE calc. values was done for each diagnosis group and model (mean, SD) s. S3 Table.

Regarding the diagnosis of severe asphyxia, the values proved identical (mean, SD) in respect to model “KHB.2016” and to “Model Matrix” and matched those of the international criteria for systemic hypothermia.

We set up plot graphs for each diagnosis group and each model to get a visual impression of the distribution of cases. In the distribution graphs the Y-axis and the X-axis represent in different combinations the values of 5- minute Apgar score, UA pH and BE per model, each graph showing the cases for all diagnoses.

**Fig 6:**
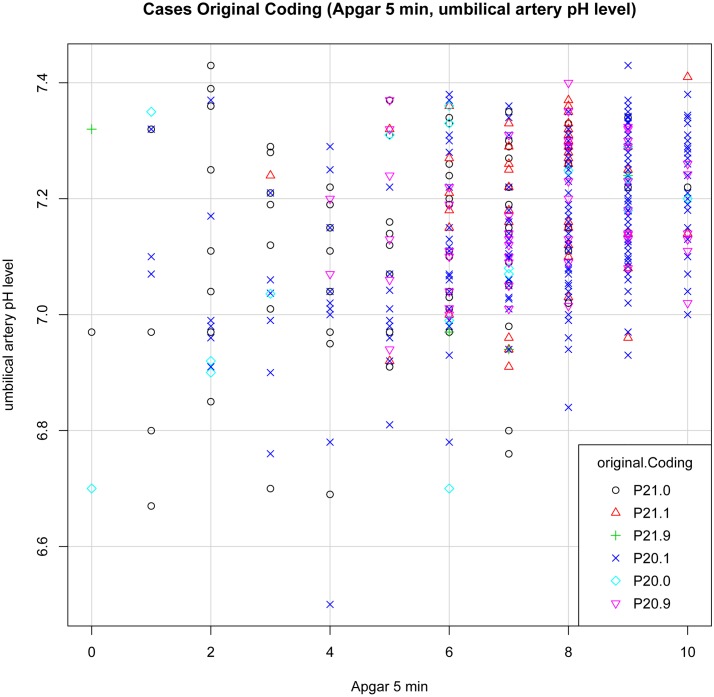
Plot Cases Original Coding (Apgar 5 min, UA pH).

Plot “Original Coding” (Fig 6): Most cases with diagnosis P21.0 can be found in the left section below. A few cases with diagnosis P20.1 show up in the area of pathological findings, but most in the area of normal clinical findings. The values do not differentiate sufficiently in respect to diagnosis.

**Fig 7:**
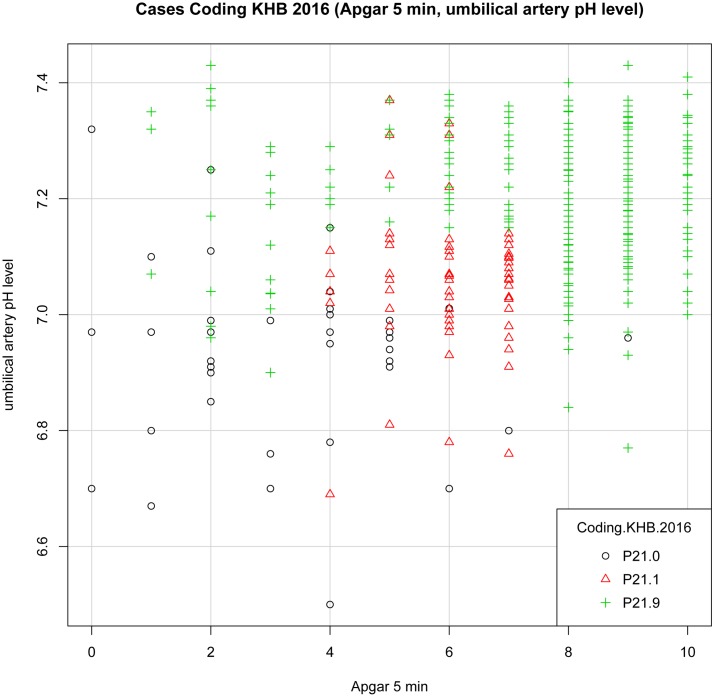
Plot Cases Coding KHB.2016 (Apgar 5 min, UA pH).

Plot “KHB.2016” (Fig 7): The cases of severe and moderate asphyxia (P21.0, P21.1) present themselves in distinct groups, but there is no clear picture concerning P21.9 “birth asphyxia unspecified”.

**Fig 8:**
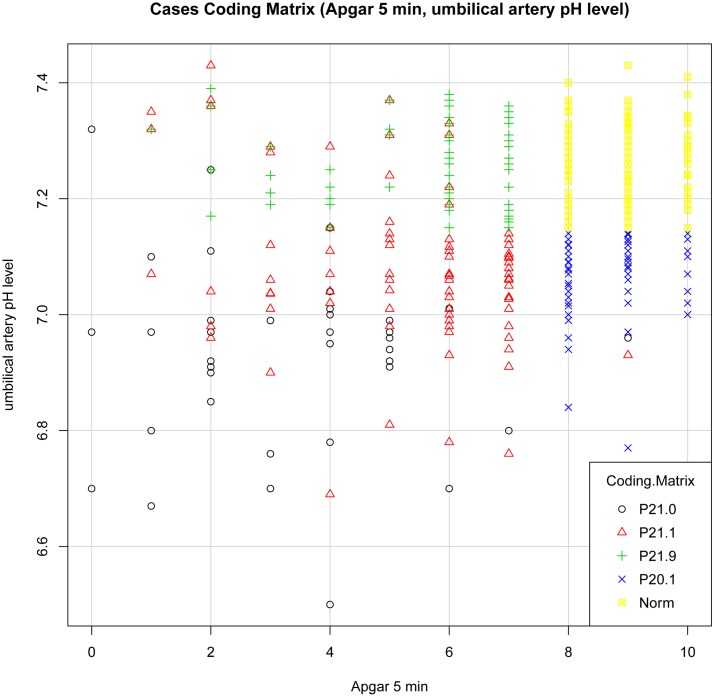
Plot Cases Coding Matrix (Apgar 5 min, UA pH).

Plot “Model Matrix” (Fig 8): A clustering of each diagnosis can be observed, but P21.1 and P21.9 appear together at a pH 7.15 and higher. The mixing of P21.1 and P21.9 might be caused by using blood pH for revision of diagnoses (values of venous or cap blood pH being lower than 7.15, the UA pH higher than 7.15).

To interpret these groups and cases, a matrix was developed which consisted of a set of two-dimensional diagrams pointing out the 3 values: 1) 5- minute Apgar score, 2) UA pH and 3) BE (S2–S4 Figs). In the distribution graphs the Y-axis represents the frequency count diagnosis and the X-axis the values of 5- minute Apgar score, UA pH and BE, each graph shows values for one certain model and diagnosis.

The diagram shows a clear distinction between values and categories and characterizes the diagnosis groups distinctly.

### Economic outcomes

The Income SwissDRG version 1.0–5.0 and the profit per SwissDRG 1.0–5.0 version were calculated for each model (“Original Coding”, “KHB.2016”, “Matrix”). To ensure the versions’ comparableness a fixed base rate of 11’000 CHF was set. Common logarithms were used to transform continuous variables to stabilize variance. Data were evaluated for F-distribution, and by visual inspection for skewness and kurtosis (histogram and QQ-diagram). The Shapiro-Wilk normality test was applied. Data points were evaluated for leverage by inspection of residuals vs. fitted plots using Cook’s D statistic. There was no normal distribution, the data were skewed. The large sample size of 622 cases permitted to use the strength of the central limit theorem of probability theory.

Profitability was evaluated using the IQR-Method [31], IQR being the difference between Quartile Q75 and Q25:

high deficit case:

Deficit<Q25−1.5×IQR

high profit case:

Profit>Q75+1.5×IQR

Financial results for each case were calculated and separated into three categories: „high deficit“, „high profit”and „normal”(„normal”meaning neither „high deficit”nor “high profit“). Cases as originally encoded and simulated according to SwissDRG version 4.0 show 46 „high profit”cases, with a total profit of 1‘427‘157.86 CHF, a mean profit per case of 31‘025 CHF (SD 16‘600 CHF). A „high deficit”was found in 64 cases, total deficit of all cases -3‘081‘090.61 CHF, mean deficit per case of -48‘142 CHF (SD 38‘936.6 CHF). The financial result of „high profit”versus „high deficit”shows a total deficit of -1‘653‘932.75 CHF. The total deficit of the billing period, of version 4.0 SwissDRG and of version 5.0 SwissDRG is -1‘380‘844.9, -2’942’591.9 and -916’838.5 CHF respectively (S4 Table).

#### Economic outcomes per coding model and diagnosis group

Total costs und log10 Costs per diagnosis and model (mean, SD) were calculated (Table 7).

**Table 7:** Log10 Costs per Diagnosis Group (mean, SD) per Model Original Coding, Coding KHB 2016, Coding Matrix.

Diagnosis	Cod.Orig (n)	Log10 Costs mean	SD	Cod.KHB.2016 (n)	Log10 Costs mean	SD	Cod. Matrix (n)	Log10 Costs mean	SD
P20.0	**22**	4.421	0.637						
P20.1	**318**	3.811	0.5397				**90**	3.752	0.475
P20.9	**59**	4.0571	0.6137						
P21.0	**145**	4.549	0.502	**81**	4.560	0.413	**80**	4.564	0.414
P21.1	**70**	4.229	0.535	**123**	4.282	0.548	**154**	4.303	0.531
P21.9	**8**	4.237	0.56	**418**	3.928	0.612	**88**	4.363	0.605
Norm							**203**	3.715	0.516
Not assigned							**7**	4.905	0.6764

Model “Original Coding”: lowest costs can be observed in the group of P20.1: mean 3.811 (SD 0.5397), similar results with the other groups: mean 4.05–4.55 (SD 0.5–0.63). Apart from group P21.0 a strong divergence of costs cannot be detected. Model „KHB.2016“: all groups show a clear tendency and distinction: P21.0, P21.1, P21.9 present declining costs. Model „Model Matrix“: Log10 costs are highest in group “not assigned”: mean 4.905 (SD 0.6764), followed by “severe asphyxia”: mean 4.564 (SD 0.414) The lowest log 10 costs appear in the group of „normal clinical finding” (mean 3.715 (SD 0.516)) and “metabolic acidosis without neurological impairment” (mean 3.752 (SD 0.475)).

#### ANOVA

**Fig 9:**
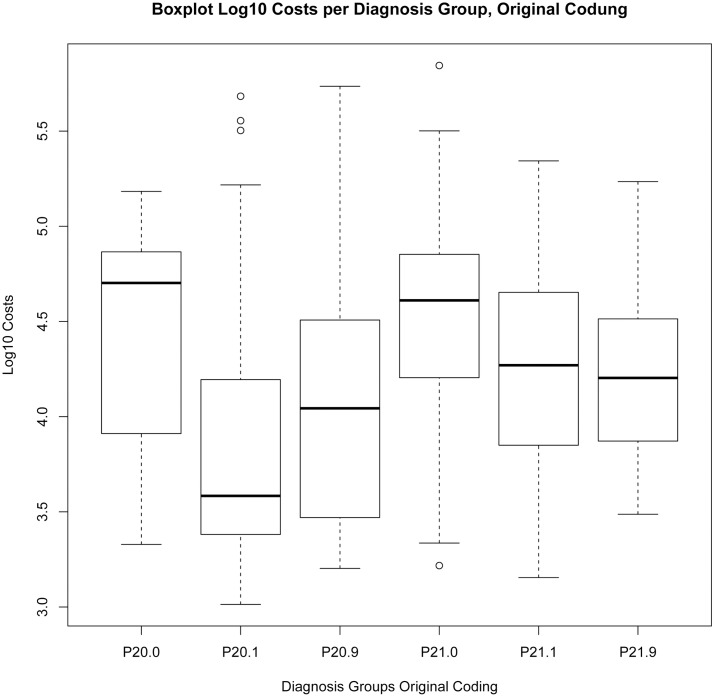
Boxplot Log10 Costs per Diagnosis Group, Original Coding.

**Fig 10:**
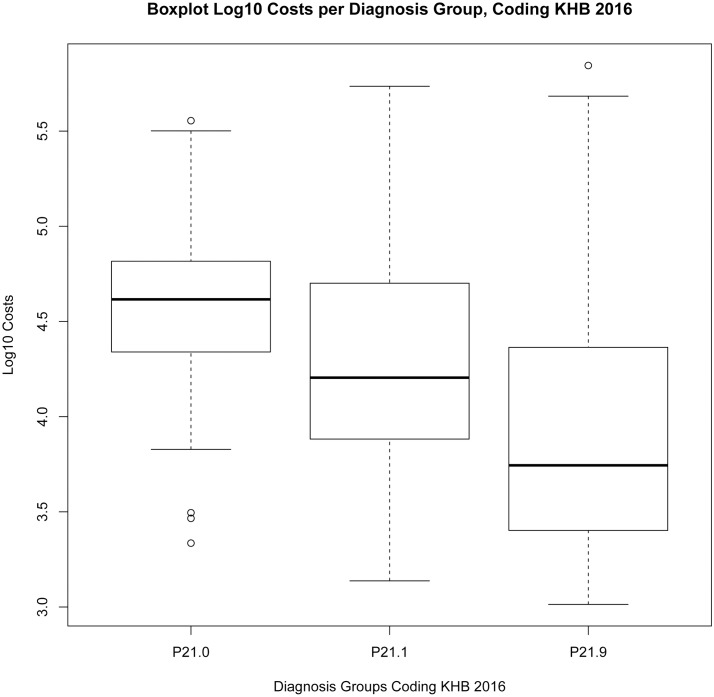
Boxplot Log10 Costs per Diagnosis Group, Coding KHB.2016.

**Fig 11:**
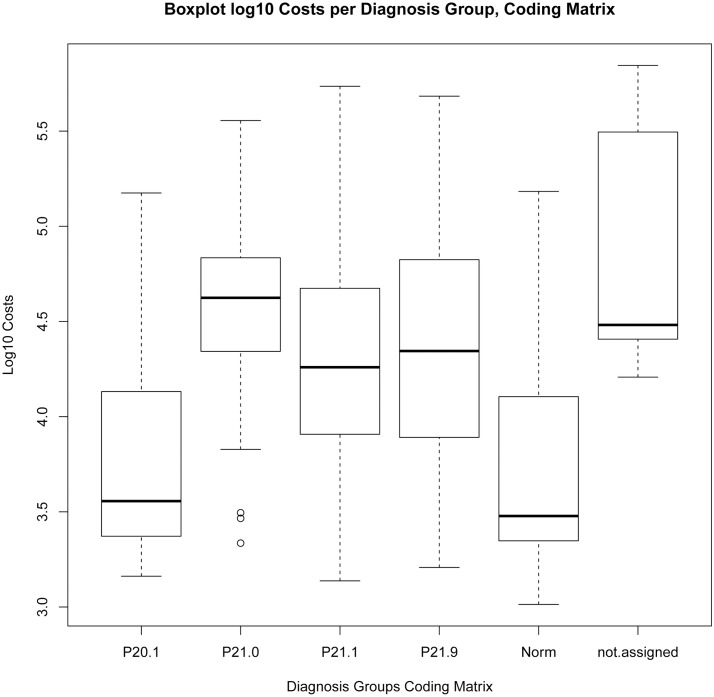
Boxplot Log10 Costs per Diagnosis Group, Coding Matrix.

The Levene-Test, the Kruskal-Wallis test and the results of the one-way independent ANOVA by comparing models “KHB.2016”, “Model Matrix” and “Original Coding” show the following results, see also Boxplot log10 costs per diagnosis group “Original Coding” Fig 9, “KHB.2016” Fig 10, “Matrix” Fig 11:

The effect of “Original Coding” encoding on the log10 Costs Variance: F(5, 616) = 40.4, p<0.0001, multiple R-squared = 0.247, adjusted R-squared = 0.2408, Levene-Test F(5, 616) = 1.0039, p>0.1; Kruskal-Wallis chi-squared (5) = 155.11, p-value < 0.001.

The effect of “KHB.2016” encoding on the Log10 Costs Variance: F(2, 619) = 50, p<0.0001, multiple R-squared = 0.1391, adjusted R-squared = 0.1363, Levene-Test F(2, 619) = 13.483, p<0.001; Kruskal-Wallis chi-squared (2) = 93.76, p < 0.001.

There was a highly significant effect of encoding according to “Model Matrix” on the log10 Costs Variance, F(5, 616) = 55.84, p<0.0001, multiple R-squared = 0.312, adjusted R-squared = 0.3063, Levene-Test F(5, 616) = 3.1798, p<0.01; Kruskal-Wallis chi-squared(5) = 197.24, p <0.001.

The distribution of costs is significantly affected by the model of encoding and its corresponding criteria. The explained variation with “Original Coding”: multiple R-squared = 0.247, adjusted R-squared = 0.2408; with “KHB.2016”: multiple R-squared = 0.1391, adjusted R-squared = 0.1363; with “Model Matrix”: multiple R-squared = 0.312, adjusted R-squared = 0.3063.

Due to the highest R-squared value “Model Matrix” gives the best explanation for cost variance.

## Discussion

Through the application of the “Model Matrix” the originally encoded diagnoses of “birth asphyxia” in 622 inpatient cases treated at the Inselspital University Hospital of Bern in between 2012–2015 were evaluated. The analysis of the clinical data presents discrepancies between the medically determined diagnosis and the ICD-10 coding which could be both identified and quantified. The hypothesis that the use of outdated ICD-10 definitions resulting in differences between the encoded diagnosis asphyxia and the medical diagnosis referring to the clinical context was confirmed.

### Indistinct ICD definitions

#### Clinical signs

Clinical signs such as „abnormal fetal heart rate“, „distress”, „meconium in liquor“, „passage of meconium” or “acidosis” are equated with ICD-10 diagnosis P20 “Intrauterine hypoxia”. Citing McLennan: „Signs of fetal compromise such as changes in fetal heart rate and passage of meconium are neither sensitive nor specific to any particular cause and only sometimes indicate damaging intrapartum hypoxia” [32]. The ACOG committee on obstetric practice warns against inappropriate use of the terms fetal distress and birth asphyxia [6].

Over the past 20 years the US National Institute of Child Health and Human Development has been elaborating terminology and interpretation of abnormal fetal heart rate [33]. However, the signs mentioned above which are associated with increased risk of neonatal encephalopathy (e.g. heart rate), show a false positive rate of 99.8 percent [34] and consequently are not rated as an equivalent diagnostic criterion for „intrauterine hypoxia”.

P20.1 “intrauterine hypoxia first noted during labour and delivery” was originally the most commonly coded diagnosis (n = 318, 51% of all cases). Relating to asphyxia signs and symptoms like meconium in liquor or abnormal pattern CTG had been documented in the patients’ records. However, these signs and symptoms are in general not specific enough to identify the diagnosis severe asphyxia, as they are only associated to 7.8% of cases [35]. Most of the patients in this group showed no apparent signs of illness and there were no cases of HIE or hypothermia treatment.

Metabolic acidosis determined solely from samples of umbilical artery at birth is a poor predictor of perinatal brain damage [26] and when associated with an Apgar Score > 7 the cases show a mostly normal outcome. 65% of cases originally encoded as P20.1 had to be mapped to „Norm”(142 cases) or to P20.1 “metabolic acidosis without neurological impairment”(64 cases).

Prepartal signs such as abnormal fetal heart rate are, if not correlated with other findings, indicators but are neither medically relevant for the diagnosis of asphyxia nor for assessing the patients’ outcome postpartum.

#### Intrapartum-related causation

The group of cases originally encoded with diagnosis P20.0 „intrauterine hypoxia before onset of labour”(22 cases) show a high variability after recoding with regard to severity ranging from diagnosis “severe asphyxia” (27% / 6 cases) to “normal clinical finding” (36% / 8 cases).

Relating facts such as a silent pattern CTG with severe asphyxia were documented in the patients‘ records, but never the term or diagnosis „intrauterine hypoxia”itself. Possible interpretations are that due to the instruction of the existing exclusion term (exclusion of P21) P20 “intrauterine hypoxia” had been encoded instead of P21 “birth asphyxia”. The ACOG, the AAP, the Task Force on Neonatal Encephalopathy and Cerebral Palsy recommend against the use of the term “birth asphyxia” unless there is clear evidence of intrapartum-related causation, as they outlined criteria which together suggest an intrapartum timing, but individually are nonspecific to asphyxia insults [9, 36]. Referring to this recommendation, the onset characteristics could be excluded from the classificatory criteria (“Model Matrix”).

#### Apgar score by 1 minute

As stated by the AAP in the “Use and Abuse of the Apgar Score” [37]: “A low 1-minute Apgar score does not correlate with the infant’s future outcome. The 5-minute Apgar score, and particularly the change in the score between 1 and 5 minutes, is a useful index of the effectiveness of resuscitation efforts”.

The 1-minute Apgar alone, listed as a defining but redundant element in the ICD diagnosis P21, should not be used as evidence of hypoxia causing neurological damage [38]. According to the Committee on Fetus and Newborn, the AAP, and the Committee on Obstetrics Practice, ACOG [39]: “An infant who has had “asphyxia” proximate to delivery that is severe enough to result in acute neurologic injury should have demonstrated all of the following criteria: (a) profound metabolic or mixed acidemia (pH < 7.00) on an umbilical arterial blood sample, if obtained, (b) an Apgar score of 0 to 3 for longer than 5 minutes, (c) neurologic manifestation, e.g, seizure, coma, or hypotonia, and (d) evidence of multiorgan dysfunction”. The 1- minute Apgar score appears to be less useful in the sense of predictability, prognosis and diagnostic accuracy than the 5- minute or 10- minute score [36, 39–49].

Our results point out, that encoding the specific diagnosis by referring to a 1- minute Apgar score of 0–3 shows a higher correlation with the medically identified diagnosis “severe asphyxia” (28%) than the 1-minute Apgar score of 4–7 (4.2%) or other elements in the ICD like the mentioned signs “meconium in liquor”, “abnormal fetal heart rate”, “distress”. However, the validity of the 1-minute Apgar score remains uncertain.

The causal relation was described by Sarnat & Sarnat in “Neonatal Encephalopathy Following Fetal Distress” [38]: “The severity of a perinatal insult is difficult to quantitate, but the postnatal course of the infant, together with EEG changes, appear to offer the best indication of later neurologic impairment”.

By the development of a new classification model (“Model Matrix”) a realistic cut-off point for defining pathological fetal acidemia which correlates with an increasing risk of neurological deficit was determined. This is defined as a pH of less than 7.00 and additionally a base deficit of more than 16 mmol/l [50, 51]. A 5-minute Apgar score as high-risk marker was used instead of the 1-minute Apgar score. The severity of perinatal neurological and metabolic impairment was outlined in opposition to etiology and time of occurrence (intrauterine long-term, intrauterine peripartum). Differentiating between an intrauterine hypoxia P20 and birth asphyxia P21 in no longer necessary.

### Applying Model Matrix

The most important characteristic of model “Model Matrix” is the possibility of classifying each individual patient based on the clinical and laboratory values and criteria into a distinct diagnosis group, regardless of level of care received or intended treatment. All these observations point out, that according to „Model Matrix”all criteria can be matched distinctly to one category. Considering the severity of illness the distribution of values seems coherent from visual perspective.

#### Severe asphyxia

The criteria of the diagnosis „severe asphyxia”P21.0 and of the indication of hypothermia treatment overlap, showing the following results (mean, SD): 5- minute Apgar 3.25, 1.92; UA pH 6.93, 0.16, UA BE -14.7, 5.16 corresponding to those of available RCT [51–53], Table 1.

80 cases were classified as severe asphyxia, 43 newborns were treated with hypothermia (68% of all hypothermia cases, n = 63).

#### Moderate asphyxia

Definition of criteria by “Model Matrix” includes 5-min Apgar score values ranging from 0 to 7 at a pH < 7.15 and at a Sarnat stage of I to II. Out of 154 newborns 15 were treated with hypothermia (23.8% of all hypothermia cases).

Including the 5- minute and 10- minute Apgar score in model “KHB.2016” appears to be advantageous and should also be considered for model “Model Matrix”, especially as the Bernese hypothermia protocol is based on the 10- minute Apgar.

#### Mild asphyxia

As there is no clearly established distinction or definition of mild versus moderate asphyxia, we defined the low cut-off value, based on review of patient records and audit case study and calculated the mean ±SD pH of all newborn between pH 7.15 and 7.20. Thus, we defined the low cut-off value at pH of 7.15 [15, 19].

It seems necessary to distinguish clearly between the diagnosis mild asphyxia and normal clinical finding. Cases with an abnormal adaptation (5- minute Apgar <7) should be allocated in group P21.9 “mild asphyxia” according to “Model Matrix”. 52% of newborns in diagnosis group P21.9 were preterm, GA < 37 WGA (46 /88), 10 of these had a birth weight < 1000 g (11% of P21.9). Due to prematurity only 3 patients of this group received systemic hypothermia treatment. The Apgar score is less reliable in premature infants, as it directly correlates with gestational age [41]. In prematurity the central nervous system reacts differently to hypoxia and symptoms of HIE manifestation present themselves less typically. The literature is not yet conclusive. Generally speaking, HIE in extremely premature infants shows a poorer outcome due to a more severe clinical state compared to on term newborn [53–59]. Therefore this group of patients might evoke a deeper interest in further analysis.

#### Normal clinical finding

Most of the patients in this group show no apparent signs of illness. Most cases (45% of „normal clinical finding“) were originally encoded in group P20.1 (142 of 318), which is due to the specified criteria such as abnormal fetal heart rate or meconium in liquor. The criteria and values set up for defining normal clinical finding in model “Model Matrix” enables coding to be more precise with respect to a distinction between normal and asphyxia. As a result of revising the cases with diagnosis P20 intrauterine hypoxia and P21 birth asphyxia according to model „Model Matrix”most cases were allocated into new groups: “normal clinical finding” (33%) and “metabolic acidosis without neurological impairment”(14%). With these two groups of patients (together 293 cases of 622, 47% of total) a clinical impact of birth hypoxia could be ruled out with a high probability. This statement could be confirmed by analyzing the patients’ records.

### Analysis of costs and reimbursement

A clear correlation between the complexity level of diagnosis and resource consumption was detected.

As could be expected in regard to resource consumption, the lowest costs were observed in the group P20.1 “Original coding”. When recoded according to „Model Matrix”the cases in group “not assigned” contribute to the highest costs followed by the cases of group P21.0 “severe asphyxia”. The lowest costs can be observed in the group of „normal clinical finding” and “metabolic acidosis without neurological impairment”.

Due to the highest R-squared value (multiple R-squared = 0.312, adjusted R-squared = 0.3063, p<0.0001) “Model Matrix” gives the best explanation for cost variance of the very heterogeneous patient population which includes neonates with a gestational age of 23–44 weeks and also congenital heart defects. Other elements such as gestational age, birth weight, ventilation, systemic hypothermia and significant OR procedures were not used for revision of cases. Although these elements are important in allocating resource consumption and costs, they were not taken into consideration in this study. The aim was to outline the relevance of diagnosis birth asphyxia in respect to reimbursement under the current DRG system.

In summary the analyses of “high deficit”and „high profit”cases point out that the 59 “high deficit” cases were responsible for 35% of all costs (7‘174‘875 CHF of 20‘612‘100 CHF). The resource consumption of certain diagnoses are not counterbalanced in the system of SwissDRG 5.0.

85.5% of cases from category „high deficit”(n = 63) belong to group P21: P21.0 (30%, 19 cases), P21.1 (28.5%, 18 cases), P21.9 (27%, 17 cases).

In version SwissDRG 5.0 (2016) the codes asphyxia P21, and severe asphyxia P21.0, are not included in any of the above mentioned mechanisms of cost allocation and consistent reimbursement, only code P20.0 “intrauterine hypoxia” is paradoxically listed as function “severe problem”. The outlined inadequacy can be explained by numerous encoding of asphyxia until 2015. We do expect a better impact of the diagnoses on the explanation of variance when encoded by validated criteria. We should now concentrate on refining the distinction of diagnoses by focusing on category P21 instead of P20. Failing to demarcate codes and diagnoses clearly and in relation to resource consumption, these interdependencies lead to an inadequate assignment of costs and resources. Our study intended to enhance development of a more sufficient DRG and comprehensive reimbursement system.

### Incidence and epidemiological research

According to Lancet Neonatal Survival Steering Team, asphyxia, one of the major direct causes of neonatal deaths globally (23% of neonatal deaths) [60], is yet difficult to determine. The diagnosis is of heterogeneous etiology, the clinical signs and symptoms are often not specific.

National statistics were used both by The Lancet Ending Preventable Stillbirths study group, The Lancet Stillbirths in High-Income Countries Investigator Group [60–65] to compare the incidence of asphyxia on an international level and by other studies for epidemiological research in perinatal medicine [3, 7, 62, 64–73].

From 2004–2014 according to figures of the Medical Statistic (Swiss Federal Statistical Office) an incidence of 40–80 cases of asphyxia per 1000 births was recorded in Switzerland, Table 2. This number exceeds the incidence in countries with a similar national neonatal mortality rate. Only the number of HIE roughly meets the expected incidence of 1.6 per 1000 live births of high-income countries. However, even with HIE the “shifting terminology and definitions of “birth asphyxia” and HIE” add specific challenges for comparability [7].

In 2005 a national cooling register for cases with hypothermia was introduced in Switzerland [20, 21, 74–77]. According to this registry the following number of neonates were treated: 2005–2010: n = 150 (mean 15 annually), 2011–2012: 121 cool and uncool (mean 60 annually). The encoded cases of the diagnosis HIE outnumber the cases of the register (by mean 100 annually). A reason for this difference has not been found yet. Although ICD codes are widely used for international statistics and research, to rely on statistics based on data of the Federal Office of Statistics bears a certain risk, especially if there is no knowledge of Swiss coding standards and coding guidelines [73]. In order to be able to submit high quality data for national and international research, the aim of a reliable and significant national statistic should be achieved.

Routinely collected health data are being increasingly used for research. Quality recommendations and standards for reporting of observational routinely-collected health data help improve the accuracy of results (STROBE, RECORD Guidelines) [78, 79].

In epidemiological research, ICD codes are being used as selection criteria. Considering in general the insufficient definitions and specifications of the ICD diagnoses, the discrepancy of incidence might be explained partly not only by health care quality but also by national coding.

### Limitations and strengths

Limitations of the study included the fact that secondary data not collected as part of our study were used. With the exception of 7 cases data of all the selected cases were complete. The methodology of data collection remained constant from 2012–2015.

Misclassification bias: No intention of upcoding, opportunistic coding and maximizing reimbursement can be observed as the diagnoses referring to asphyxia are irrelevant for DRG classification. Furthermore, encoded cases are revised annually and systematic in-house quality checks are performed.

It has to be taken into account, that any change in definitions concerning code P20.1 may cause adjustments in practice of encoding, statistics and reimbursement. We acknowledge that the discrepancy of encoded diagnosis and medically determined diagnosis is increased by the exclusion term and by the existing definition in code P20 and should be revised.

Missing variables: Information on laboratory findings of outborn patients should be obtained, the process has to be improved. But most important, the relevant biochemical values in complex cases were all registered.

Our study has several strengths. We analyzed standardized data of all inpatients of our hospital, our results providing indices for university hospitals in general, to our knowledge, a unique approach in Swiss research. As the criteria can be verified, the refined model “Model Matrix” offers the advantage of being able to calculate the PPV (positive predictive value), NPV (negative predictive value), TP (true positive), TN (true negative) (79) with respect to diagnosis.

## Conclusions

There had been extensive encoding efforts of asphyxia from 2012 until 2015. To achieve a reasonable progress concerning the SwissDRG system, quality of data must be improved. This requires an accurate diagnosis as well as corresponding coding guidelines.

Through the definition of five diagnosis groups, a distinct allocation of cases can be achieved. The newly introduced model “Model Matrix” (Apgar score, Sarnat stage, pH, BE) explains approximately 30% of cost variance of a very heterogeneous group of patients and appears highly suitable for clinical use, research and reimbursement.

## Supporting Information

S1 Fig Coding Guidelines 2016. (TIFF)

S2 Fig Plot “Original Coding”. (TIFF)

S3 Fig Plot “Coding.KHB”. (TIFF)

S4 Fig Plot “Matrix”. (TIFF)

S1 Table Number of Diagnoses P20*, P21*, P91* coded in Switzerland for 2004–2014. (DOCX)

S2 Table Number of live births in Switzerland in 2004–2014. (DOCX)

S3 Table Biochemical Values Cases. (DOCX)

S4 Table Earning SwissDRG billing Year. (DOCX)

S1 File CHOP Codes which indicated a mechanical ventilation, a systemic hypothermia and significant OR procedure. (DOCX)

S1 Data Raw data underlying the findings. (XLSX)
