# Supplementary figures and images for: Asphyxia in the Newborn: Evaluating the Accuracy of ICD Coding, Clinical Diagnosis and Reimbursement: Observational Study at a Swiss Tertiary Care Center on Routinely Collected Health Data from 2012-2015
Source: PLoS One. 2017 Jan 24;12(1):e0170691. doi: 10.1371/journal.pone.0170691 (PMC5261744; doi:10.1371/journal.pone.0170691)

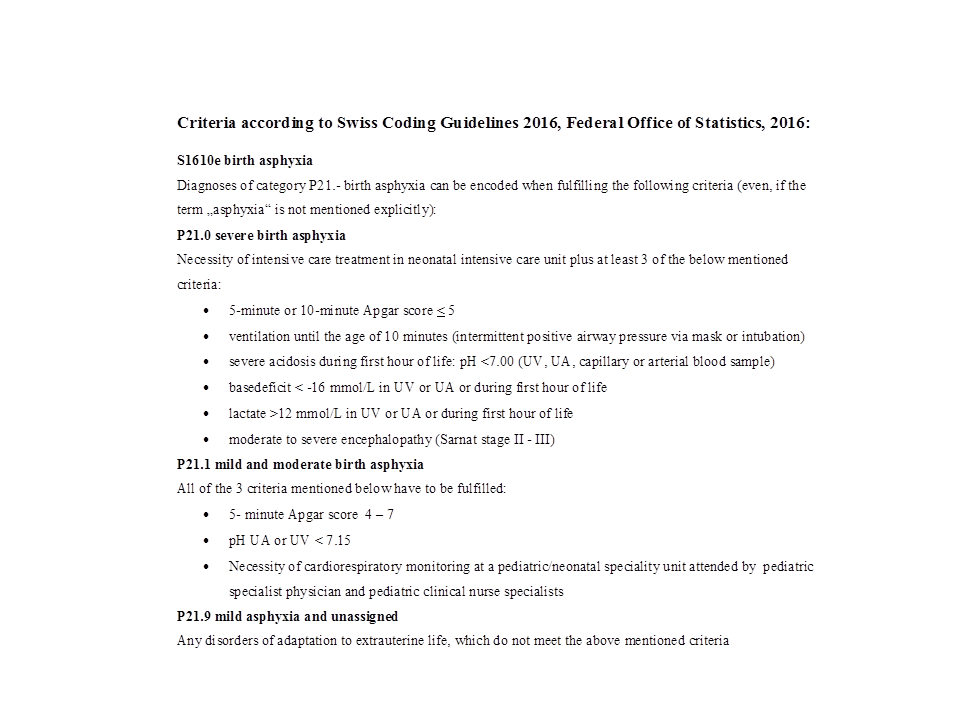

Supplement: S1 Fig — (TIFF) [file pone.0170691.s001.TIFF]

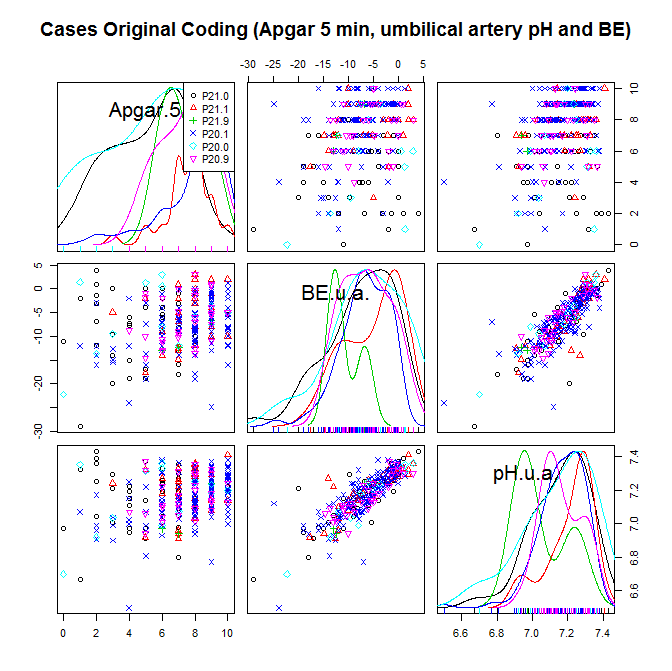

Supplement: S2 Fig — (TIFF) [file pone.0170691.s002.tiff]

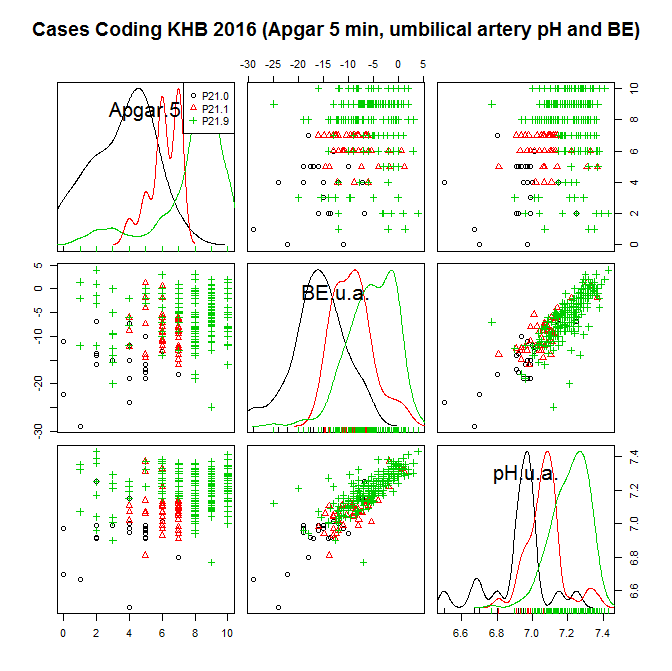

Supplement: S3 Fig — (TIFF) [file pone.0170691.s003.tiff]

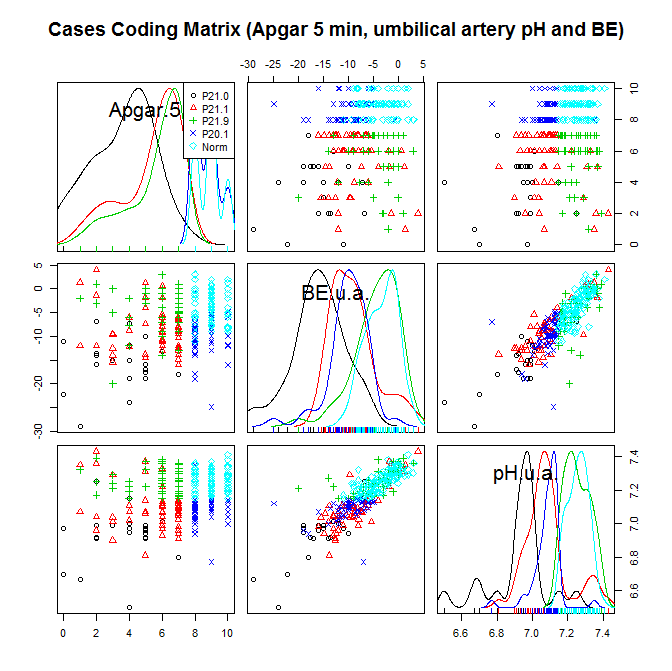

Supplement: S4 Fig — (TIFF) [file pone.0170691.s004.tiff]
