# Supplementary material for: Asphyxia in the Newborn: Evaluating the Accuracy of ICD Coding, Clinical Diagnosis and Reimbursement: Observational Study at a Swiss Tertiary Care Center on Routinely Collected Health Data from 2012-2015
Source: PLoS One. 2017 Jan 24;12(1):e0170691. doi: 10.1371/journal.pone.0170691 (PMC5261744; doi:10.1371/journal.pone.0170691)
Supplement: S1 Table — (DOCX) [file pone.0170691.s005.docx]

S1 Table. Number of Diagnoses P20*, P21*, P91* coded in Switzerland for 2004 – 2014, Medical Statistics, Swiss Federal Statistical Office.

| **Diagnosis or procedure** | **Year** | | | | | | | | | | |
| --- | --- | --- | --- | --- | --- | --- | --- | --- | --- | --- | --- |
|  | **2004** | **2005** | **2006** | **2007** | **2008** | **2009** | **2010** | **2011** | **2012** | **2013** | **2014** |
| **P20* and/or P21* Intrauterine hypoxia and/or birth asphyxia** | 4009 | 5788 | 5685 | 5942 | 5831 | 5556 | 4704 | 4801 | 4658 | 4064 | 3771 |
| **P20* and P21* Intrauterine hypoxia and birth asphyxia (the same case)** | 401 | 551 | 421 | 386 | 377 | 447 | 264 | 272 | 240 | 193 | 100 |
| **P20.0 Intrauterine hypoxia, first noted before onset of labour** | 100 | 106 | 148 | 137 | 125 | 137 | 101 | 125 | 82 | 80 | 67 |
| **P20.1 Intrauterine hypoxia first noted during labour and delivery** | 1780 | 2632 | 2428 | 1916 | 1836 | 2300 | 1528 | 1432 | 1802 | 2042 | 2384 |
| **P20.9 Intrauterine hypoxia, unspecified** | 472 | 528 | 402 | 474 | 297 | 266 | 205 | 226 | 261 | 207 | 272 |
| **P21.0 Severe birth asphyxia** | 336 | 464 | 465 | 556 | 662 | 654 | 640 | 680 | 684 | 586 | 480 |
| **P21.1 Mild and moderate birth asphyxia** | 1165 | 1897 | 2101 | 2256 | 2322 | 2100 | 2136 | 2250 | 1781 | 1089 | 568 |
| **P21.9 Birth asphyxia, unspecified** | 156 | 161 | 141 | 603 | 589 | 99 | 94 | 88 | 48 | 60 | 43 |
| **P91* Other disturbances of cerebral status of newborn** | 12 | 28 | 26 | 29 | 39 | 104 | 109 | 131 | 165 | 149 | 182 |
| **P91.6 Hypoxic-ischemic encephalopathy of newborn** |  |  |  |  |  | 78 | 59 | 87 | 95 | 84 | 102 |
| **P91.3 Neonatal cerebral irritability / P91.3^a^ and P91.6 Neonatal cerebral irritability by HIE^b^** | 6 | 17 | 14 | 15 | 23 | 21 | 22/5 | 19/7 | 34/24 | 24/17 | 32/18 |
| **P91.4 Neonatal cerebral depression / P91.4^c^ and P91.6 Neonatal cerebral depression by HIE** | 2 | 4 | 4 | 8 | 10 | 5/1 | 25/14 | 19/14 | 25/22 | 27/21 | 28/19 |
| **P91.5 Neonatal coma / P91.5^d^ and P91.6 Neonatal coma by HIE** | 4 | 7 | 8 | 6 | 6 | 0 | 3/3 | 6/4 | 11/9 | 14/13 | 20/17 |
| **(P20* and /or P21*) and P91*^e^** | 27 | 34 | 55 | 72 | 64 | 82 | 83 | 102 | 95 | 114 | 114 |
| **(P20*, P21* or P91*)^f^ and Procedure 99.81.20^f, g^** |  |  |  |  |  |  |  | 51 | 35 | 51 | 68 |
| P20*, P21*, P91* tree characters Code  ^a^ P91.3, Neonatal cerebral irritability corresponds HIE I (Swiss Coding Guidelines)  ^b^ HIE, hypoxic-ischemic encephalopathy  ^c^P91.4, Neonatal cerebral depression corresponds HIE II (Swiss Coding Guidelines)  ^d^P91.5, Neonatal coma corresponds HIE III  ^e^ The combination (P20* and/or P21*) and P91* as equivalent of “HIE by intrauterine hypoxia and/or birth asphyxia”  ^f^ 99.81.20 procedure code for systemic hypothermia (CHOP, Swiss operations and procedures catalog)  ^g^ The (P20*, P21* or P91*)f and procedure 99.81.20 as equivalent of “Hypothermia by intrauterine hypoxia, birth asphyxia or disturbance of cerebral status of newborn”. P20*, P21* or P91* only as a main diagnosis in the newborn, age by admission < 6 days. | | | | | | | | | | | |
