# Supplementary material for: Asphyxia in the Newborn: Evaluating the Accuracy of ICD Coding, Clinical Diagnosis and Reimbursement: Observational Study at a Swiss Tertiary Care Center on Routinely Collected Health Data from 2012-2015
Source: PLoS One. 2017 Jan 24;12(1):e0170691. doi: 10.1371/journal.pone.0170691 (PMC5261744; doi:10.1371/journal.pone.0170691)
Supplement: S2 Table — (DOCX) [file pone.0170691.s006.docx]

S2 Table. Number of live births in Switzerland in 2004 – 2014, Swiss Federal Statistical Office.

| **Number of live births, Statistics** | **Year** | | | | | | | | | | |
| --- | --- | --- | --- | --- | --- | --- | --- | --- | --- | --- | --- |
|  | **2004** | **2005** | **2006** | **2007** | **2008** | **2009** | **2010** | **2011** | **2012** | **2013** | **2014** |
| MS^a^ | 71430 | 72193 | 72946 | 73989 | 76212 | 77690 | 80508 | 80646 | 82607 | 83098 | 85234 |
| BEVNAT^b^ | 73082 | 72903 | 73371 | 74494 | 76691 | 78286 | 80290 | 80808 | 82164 | 82731 | 85287 |
| ^a^The number of live births according MS (Medical Statistics of the Hospitals): included only the births in hospital-setting ^b^The number of live births according BEVNAT Statistics, included all births. From 2010 new definition of the permanent resident population, which also includes asylum seekers with a total length of stay of at least 12 months | | | | | | | | | | | |
