# Supplementary material for: Asphyxia in the Newborn: Evaluating the Accuracy of ICD Coding, Clinical Diagnosis and Reimbursement: Observational Study at a Swiss Tertiary Care Center on Routinely Collected Health Data from 2012-2015
Source: PLoS One. 2017 Jan 24;12(1):e0170691. doi: 10.1371/journal.pone.0170691 (PMC5261744; doi:10.1371/journal.pone.0170691)
Supplement: S3 Table — (DOCX) [file pone.0170691.s007.docx]

**S3 Tables 1 -8, Values Apgar, pH, BE by diagnosis group and model**.

**Table 1. Apgar by age of 5 min by diagnosis groups original coding.**

| **Apgar by 5 min**  **Original Coding** | **Mean** | **SD** | **Min** | **Max** | **n** | **Value missing** |
| --- | --- | --- | --- | --- | --- | --- |
| **P20.0** | 5.86 | 2.91 | 0 | 10 | 22 | 0 |
| **P20.1** | 7.56 | 2.11 | 1 | 10 | 316 | 2 |
| **P20.9** | 7.39 | 1.71 | 3 | 10 | 58 | 1 |
| **P21.0** | 4.62 | 2.35 | 0 | 10 | 144 | 1 |
| **P21.1** | 7.47 | 1.2 | 3 | 10 | 69 | 1 |
| **P21.9** | 5.62 | 2.82 | 0 | 9 | 8 | 0 |

**Table 2. Umbilical artery pH by diagnosis groups original coding.**

| **UA pH**  **Original Coding** | **Mean** | **SD** | **Min** | **Max** | **n** | **Value missing** |
| --- | --- | --- | --- | --- | --- | --- |
| **P20.0** | 7.11 | 0.21 | 6.7 | 7.36 | 17 | 5 |
| **P20.1** | 7.16 | 0.13 | 6.5 | 7.44 | 263 | 55 |
| **P20.9** | 7.16 | 0.11 | 6.94 | 7.4 | 51 | 8 |
| **P21.0** | 7.13 | 0.17 | 6.67 | 7.43 | 77 | 68 |
| **P21.1** | 7.21 | 0.13 | 6.91 | 7.41 | 49 | 21 |
| **P21.9** | 7.11 | 0.19 | 6.94 | 7.32 | 4 | 4 |
| UA, umbilical artery | | | | | | |

**Table 3. Umbilical artery BE by diagnosis groups original coding.**

| **UA BE**  **Original Coding** | **Mean** | **SD** | **Min** | **Max** | **n** | **Value missing** |
| --- | --- | --- | --- | --- | --- | --- |
| **P20.0** | -5.35 | 6.73 | -22.3 | 3 | 16 | 6 |
| **P20.1** | -6.57 | 5.03 | -25.0 | 2 | 178 | 140 |
| **P20.9** | -6.5 | 4.76 | -14.6 | 3 | 35 | 24 |
| **P21.0** | -7.39 | 6.61 | -29.0 | 4 | 66 | 79 |
| **P21.1** | -4.18 | 5.41 | -17.7 | 3.1 | 50 | 20 |
| **P21.9** | -10.7 | 3.47 | -13.0 | -6.7 | 3 | 5 |
| UA, umbilical artery; BE, base excess | | | | | | |

**Table 4. Apgar by age of 5 min by diagnosis groups coding KHB 2016.**

| **Apgar 5 min**  **Coding KHB2016** | **Mean** | **SD** | **Min** | **Max** | **n** | **Value missing** |
| --- | --- | --- | --- | --- | --- | --- |
| **P21.0** | 3.3 | 1.95 | 0 | 9 | 81 | 0 |
| **P21.1** | 6 | 1.01 | 4 | 7 | 123 | 0 |
| **P21.9** | 7.67 | 2.1 | 1 | 10 | 413 | 5 |

**Table 5. Umbilical artery pH by diagnosis groups coding KHB 2016.**

| **UA pH**  **Coding KHB2016** | **Mean** | **SD** | **Min** | **Max** | **n** | **Value missing** |
| --- | --- | --- | --- | --- | --- | --- |
| **P21.0** | 6.93 | 0.16 | 6.5 | 7.32 | 37 | 44 |
| **P21.1** | 7.05 | 0.11 | 6.69 | 7.37 | 79 | 44 |
| **P21.9** | 7.21 | 0.11 | 6.77 | 7.44 | 345 | 73 |
| UA, umbilical artery | | | | | | |

**Table 6. Umbilical artery BE by diagnosis groups coding KHB 2016.**

| **UA BE**  **Coding KHB2016** | **Mean** | **SD** | **Min** | **Max** | **n** | **Value missing** |
| --- | --- | --- | --- | --- | --- | --- |
| **P21.0** | -14.7 | 5.16 | -29 | -2 | 32 | 49 |
| **P21.1** | -7.93 | 4.35 | -16 | 1.3 | 64 | 59 |
| **P21.9** | -4.9 | 2.1 | -25 | 4 | 252 | 166 |
| UA, umbilical artery; BE, base excess | | | | | | |

**Table 7. Apgar by age of 5 min by diagnosis groups coding Matrix.**

| **Apgar 5 min**  **Coding Matrix** | **Mean** | **SD** | **Min** | **Max** | **n** | **Value missing** |
| --- | --- | --- | --- | --- | --- | --- |
| **P20.1** | 8.65 | 0.7 | 8 | 10 | 90 |  |
| **P21.0** | 3.25 | 1.92 | 0 | 9 | 80 |  |
| **P21.1** | 5.47 | 1.70 | 1 | 10 | 154 |  |
| **P21.9** | 5.69 | 1.66 | 1 | 7 | 88 |  |
| **Norm** | 8.8 | 0.68 | 8 | 10 | 202 | 1 |
| **Not assigned** | 5 | 3.6 | 2 | 9 | 3 | 4 |

**Table 8. Umbilical artery pH by diagnosis groups coding Matrix.**

| **UA pH**  **Coding Matrix** | **Mean** | **SD** | **Min** | **Max** | **n** | **Value missing** |
| --- | --- | --- | --- | --- | --- | --- |
| **P20.1** | 7.08 | 0.07 | 6.77 | 7.35 | 83 | 7 |
| **P21.0** | 6.93 | 0.16 | 6.50 | 7.32 | 37 | 43 |
| **P21.1** | 7.07 | 0.13 | 6.69 | 7.43 | 101 | 53 |
| **P21.9** | 7.25 | 0.07 | 7.15 | 7.39 | 68 | 20 |
| **Norm** | 7.23 | 0.062 | 7.15 | 7.43 | 171 | 32 |
| **Not assigned** | 7.44 | na | 7.44 | 7.44 | 1 | 6 |
| UA, umbilical artery | | | | | | |

**Table 9. Umbilical artery BE by diagnosis groups coding KHB 2016.**

| **UA BE**  **Coding Matrix** | **Mean** | **SD** | **Min** | **Max** | **n** | **Value missing** |
| --- | --- | --- | --- | --- | --- | --- |
| **P20.1** | -9.95 | 3.95 | -25 | -0.6 | 51 | 39 |
| **P21.0** | -14.7 | 5.16 | -29 | -2.0 | 32 | 48 |
| **P21.1** | -7.92 | 4.77 | -16 | 4.0 | 77 | 77 |
| **P21.9** | -3.53 | 4.43 | -20 | 3.0 | 60 | 28 |
| **Norm** | -3.21 | 3.19 | -11 | 3.1 | 128 | 75 |
| **Not assigned** | na | na | na | na | 0 | 7 |
| UA, umbilical artery; BE, base access | | | | | | |
