# Supplementary material for: Asphyxia in the Newborn: Evaluating the Accuracy of ICD Coding, Clinical Diagnosis and Reimbursement: Observational Study at a Swiss Tertiary Care Center on Routinely Collected Health Data from 2012-2015
Source: PLoS One. 2017 Jan 24;12(1):e0170691. doi: 10.1371/journal.pone.0170691 (PMC5261744; doi:10.1371/journal.pone.0170691)
Supplement: S4 Table — (DOCX) [file pone.0170691.s008.docx]

S4 Table. Earning SwissDRG simulated for Coding KHB 2016 and Matrix.

| Income, CHF | Total, (n=622) | Earning, CHF |
| --- | --- | --- |
| Earning SwissDRG billing period, simulated^a^, Original Coding | 19‘231‘256 | -1‘380‘844.9 |
| Earning SwissDRG 4.0, Original Coding | 17’669’509 | -2’942’591.9 |
| Earning SwissDRG 5.0, Original Coding | 19’695’262.4 | -916’838.5 |
| Earning SwissDRG 4.0, Coding KHB 2016 | 17’681’015 | -2’931’085.9 |
| Earning SwissDRG 5.0, Coding KHB 2016 | 19’654’013.5 | -958’087.4 |
| Earning SwissDRG 4.0, Coding Matrix | 17’662’689 | -2’949’411.9 |
| Earning SwissDRG 5.0, Coding Matrix | 19’643’618.5 | -968’482.4 |
| ^a^ All simulation were performed with base rate 11’000 CHF for better comparability | | |
