# Supplementary material for: Asphyxia in the Newborn: Evaluating the Accuracy of ICD Coding, Clinical Diagnosis and Reimbursement: Observational Study at a Swiss Tertiary Care Center on Routinely Collected Health Data from 2012-2015
Source: PLoS One. 2017 Jan 24;12(1):e0170691. doi: 10.1371/journal.pone.0170691 (PMC5261744; doi:10.1371/journal.pone.0170691)
Supplement: S1 File — (DOCX) [file pone.0170691.s009.docx]

S1 File. **CHOP Codes which indicated a mechanical ventilation, a systemic hypothermia and significant OR procedure**

1. CHOP codes which indicated a mechanical ventilation: 96.A1.10 “Mechanische Beatmung Neugeborener und Säuglinge” (2012, 2013, 2014); 96.A1.12 „Mechanische Beatmung Neugeborener und Säuglinge“ (2015).
2. CHOP coeds which indicated a systemic hypothermia: 99.81.20 “Systemische Hypothermie” (2012 – 2015)
3. CHOP codes which indicate significant OR procedures MDC 15:

2012

<http://swissdrg.org/assets/pdf/System_10/DefHandbuch_SwissDRG_1_0_Abrechnungsversion_Band3.pdf>

2013

<http://swissdrg.org/assets/pdf/System_20/DefHandbuch_SwissDRG_2_0_Abrechnungsversion_Band3.pdf>

2014

<http://swissdrg.org/assets/pdf/System_30/DefHandbuch_SwissDRG_3_0_Abrechnungsversion_Band3.pdf>

2015

<http://swissdrg.org/assets/pdf/System_40/Abrechnungsversion_40/DefHandbuch_SwissDRG_4_0_Abrechnungsversion_Band3.pdf>
